# Another One Bites the Gut: Nuclear Receptor LRH-1 in Intestinal Regeneration and Cancer

**DOI:** 10.3390/cancers13040896

**Published:** 2021-02-20

**Authors:** Roberta Zerlotin, Maria Arconzo, Elena Piccinin, Antonio Moschetta

**Affiliations:** 1 Department of Interdisciplinary Medicine, University of Bari “Aldo Moro”, 70124 Bari, Italy; roberta.zerlotin@uniba.it (R.Z.); maria.arconzo@uniba.it (M.A.); elena.piccinin@uniba.it (E.P.); 2 Department of Basic Medical Sciences, Neurosciences and Sense Organs, University of Bari “Aldo Moro”, 70124 Bari, Italy; 3 INBB, National Institute for Biostructures and Biosystems, 00136 Rome, Italy; 4 National Cancer Center, IRCCS Istituto Tumori Giovanni Paolo II, 70124 Bari, Italy

**Keywords:** intestine, intestinal stem cells (ISC), inflammatory bowel disease (IBD), colorectal cancer (CRC), nuclear receptors (NR), liver receptor homolog 1 (LRH-1)

## Abstract

**Simple Summary:**

The nuclear receptor Liver Receptor Homolog-1 (LRH-1) is widely involved in the complex and balanced biology of the intestine, thus guaranteeing the several functions played by this organ. Alterations of LRH-1 pathways are involved in tumor formation. This review covers the main aspects related to LRH-1 contribution in both physiological and pathological aspects of the intestine.

**Abstract:**

The process of self-renewal in normal intestinal epithelium is characterized by a fine balance between proliferation, differentiation, migration, and cell death. When even one of these aspects escapes the normal control, cellular proliferation and differentiation are impaired, with consequent onset of tumorigenesis. In humans, colorectal cancer (CRC) is the main pathological manifestation of this derangement. Nowadays, CRC is the world’s fourth most deadly cancer with a limited survival after treatment. Several conditions can predispose to CRC development, including dietary habits and pre-existing inflammatory bowel diseases. Given their extraordinary ability to interact with DNA, it is widely known that nuclear receptors play a key role in the regulation of intestinal epithelium, orchestrating the expression of a series of genes involved in developmental and homeostatic pathways. In particular, the nuclear receptor Liver Receptor Homolog-1 (LRH-1), highly expressed in the stem cells localized in the crypts, promotes intestine cell proliferation and renewal in both direct and indirect DNA-binding manner. Furthermore, LRH-1 is extensively correlated with diverse intestinal inflammatory pathways. These evidence shed a light in the dynamic intestinal microenvironment in which increased regenerative epithelial cell turnover, mutagenic insults, and chronic DNA damages triggered by factors within an inflammatory cell-rich microenvironment act synergistically to favor cancer onset and progression.

## 1. Introduction

Colorectal cancer (CRC) is the third most common tumor and the second leading cause of cancer death in the world (Globocan 2020, https://gco.iarc.fr/, accessed on 20 December 2020) [1]. Recently, it has been established that the onset of CRC in young subjects is increased, and the number of cases will raise by about 90% in the United States in 2030 [2]. In many cases, CRC may evolve from a pre-existing condition of intestinal inflammation [3]. Although the main mechanisms by which inflammation can lead to tumor development are still to be uncovered, the strict connection between inflammation and cancer is every day more persuasive. Until now, different metabolic and molecular pathways have been investigated to find specific targets that may encourage the development of selective therapy able to counteract the intestinal inflammation and eventually block the progression to severe forms of diseases, including cancer. Nuclear receptors (NRs) have historically been a crucial topic of cancer research, by integrating both metabolic and molecular processes [4].

NRs are a superfamily of ligand-activated transcription factors, which orchestrate molecular events that direct a complex regulatory expression network, thus resulting in the fine-tunning of physiological and pathological processes [5]. Several NRs and related coregulators have been so far individuated, which collectively act by sensing the intracellular changes in hormones or metabolites and by eliciting a specific response that results in the expression of downstream target genes [6]. Among these NRs, the Liver Receptor Homolog 1 (LRH-1) has been characterized for its peculiar properties involved in the stem cells regulation, in the inflammation response as well as in the metabolic functions, which allow the development and the homeostasis of several tissues, especially those of endodermic derivation [7]. The organ that better recapitulate all the functions played by LRH-1 is probably the intestine, where this NR is indispensable for the maintenance of a healthy gut epithelium. Indeed, altering LRH-1 expression results in the exacerbation of intestinal inflammation that may finally culminates in CRC onset and progression [8].

To understand the varied and multifaceted role of LRH-1, and how perturbance on its pathway may lead to and sustain CRC, we firstly shed a light on the intestinal architecture, underlining the key contribution of stem cell residing in the crypt compartment not only to the maintenance of gut homeostasis but also to tumor development. Moreover, we analyze the potential contribution of intestinal inflammation that may represent a continuum to CRC onset. Finally, we dissect the functions of LRH-1 in the preservation of intestinal homeostasis, in the inflammatory bowel diseases, and in cancer conditions.

## 2. The Intestine: A Well-Organized Architecture behind Multiple Functions

The intestine retains a peculiar architecture, which allow several physiological functions, including digestion, absorption of nutrients, secretion and absorption of electrolytes and water, as well as defense against pathogens and noxious agents.

The most peculiar layer of the intestine is the mucosa characterized by the presence of millions of crypt-villus units [9], unique structures assuring the fulfillment of the whole intestinal functions. The villi are finger-like protrusions facing the lumen of the organ, found specifically in the small intestine. They are covered by enterocytes forming a highly selective barrier that acts as a shield between the host and the intestinal lumen. The enterocytes show on their apical membrane numerous short finger extensions, the microvilli, contributing to further increase the absorption surface. Interestingly, the villus length progressively decreases from the duodenum towards the ileum and consequently the absorption function gradually reduces in the last area of small intestine [10]. In contrast, the epithelial surface of the large intestine is essentially flat, reflecting the fact that the colon has a major role in stool compaction rather than absorption [11].

**Figure 1:**
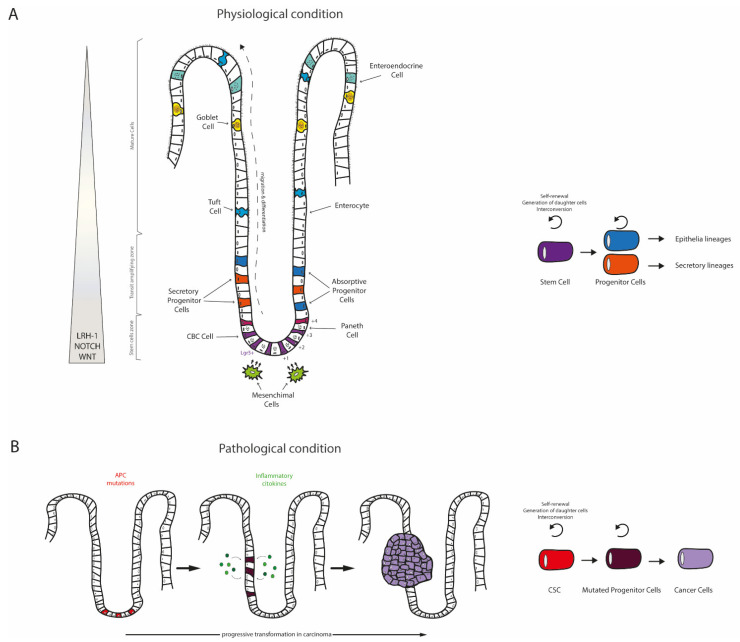
The intestine under physiological and pathological conditions. The small intestine presents a well-organized architecture, composed by villi surrounded by multiple crypts. The mucosa of large intestine lacks villi and shows only crypts. These structures are completely disrupted during cancer. (**A**) In physiological condition, the crypt based columnar (CBC) cells act as stem cells, expressing Lgr5 and generating a continuous flow of cells that move toward the tip. Notably, the main active pathways in the stem cell zone are those of WNT and NOTCH, which are strictly regulated by the expression of the Liver Receptor Homolog-1 (LRH-1). This scenario is present both in the small and in the large intestine. Paneth cells surround and metabolically sustain CBC cells located in position +1 to +3. In addition, mesenchymal cells can secrete factors that nurture the stem cells in the crypt. In the transit amplifying zone, progenitor cells divide quickly, in order to sustain the rapid cell renewal of the intestine, and differentiate in secretory (Tuft, Goblet, and enteroendocrine cells) or absorptive (enterocytes) lineages. (**B**) The colorectal cancer (CRC) can be caused by repetitive insults to the intestinal cells. The first event usually involves alterations to the WNT/β-catenin pathway, followed by inflammatory cytokines exposition that drive cancer development. Notably, the cells responsible of tumor initiation and progression are the cancer stem cells (CSC) that retain characteristic of stem cells and allow an undefined and incessant cell growth together with the ability to invade distant tissues, promoting metastases.

Regular short introflexions of intestinal epithelium, called the crypts of Lieberkühn, are present in both small and large intestine. The crypt environment is isolated from the intestinal lumen, thus guaranteeing the undisturbed proliferation and differentiation of stem cells located on the bottom of the crypt, the so called “crypt base columnar cells” (CBC). These cells can be considered as adult intestinal stem cells, since they allow the daily renewal of damaged epithelial cells facing the intestinal lumen, therefore contributing to the maintenance of the gut functionality [12]. Thanks to CBC, the intestinal epithelium is replaced every 3–4 days in mice and every week in human [13,14], thereby resulting the most actively regenerating tissue of the mammalian ones. Particularly, CBC give rise to proliferative progenitor cells, which can further differentiate into absorptive (enterocytes) or secretory (goblet, Tuft, Paneth, and enteroendocrine cells) lineages. Once the progenitors differentiate into enterocytes, these mature cells move from the bottom to the top of the crypt [13,15]. This ability of unceasing proliferation and differentiation coupled with the migration along the crypt–villus axis ensures the perfect efficiency of the intestinal epithelium. Potential modifications of the epithelial renewal compromising the integrity of the intestinal barrier are involved in pathological situations that lead to the alterations of tissue homeostasis, inflammation, and tumor development [16]. Besides the enterocytes, other mature cells are found inside the crypt. These cells belong to the secretory cell lineages and comprise mucus-producing goblet cells, important for the removal of potentially dangerous contaminants, pathogens, and microbial products (toxins), finally protecting the intestinal cells [17,18]. Other mature secretory cells in the crypt are the enteroendocrine cells, which secrete hormones such as serotonin or secretin [19]; the Paneth cells, which release antimicrobial peptides and enzymes such as defenses, cryptidins, and lysozyme [20,21]; and the Tuft cells, serving immune regulatory function (Figure 1A) [22,23].

In physiological conditions, the WNT pathway is crucial for the maintenance of stem cells and their differentiation. Indeed, alterations of this pathway can lead to several pathological conditions, including cancer [24]. WNT pathway is activated by WNT ligands, which bind to Frizzled receptor and LRP5/6 coreceptor. The formation of this trimeric complex leads to inhibition of β-catenin destruction by cytoplasmic adenomatous polyposis coli (APC) destruction complex. In this way, stabilized β-catenin can translocate to the nucleus, where it associates with members of the TCF family of transcription factors (TCF1, LEF, TCF3, and TCF4) [25]. Clevers and colleagues highlighted that among the genes targeted by WNT signaling, the leucine-rich-repeat-containing G-protein-coupled receptor 5 (LGR5) was restrictively expressed in CBCs in the small intestine and in the colon [26]. LGR5+ cells are exclusively present at the bottom of the crypt, assuring multiple cells renewal and generation of multiple lineages. Therefore, LGR5 has been considered as the most prominent marker of intestinal stem cells. Although the pivotal importance of WNT signal in self-renewal of intestinal epithelium, the activation of other pathways is also linked to the regenerative properties of the intestinal mucosa [27], such as Notch and YAP/TAZ pathway [28]. Moreover, many molecules (WNT ligands, EGF, R-spondin, DLL4, subepithelial mesenchymal GLI1, and PDGFR-α) produced in the stem cells environment crucially support the LGR5+ stem cell metabolism [29,30,31,32,33,34]. Altogether, it is clear that the multiple levels of interaction between cells and signals from both inside and outside the crypt are required to protect and nurture the intestinal stem cells, thus promoting a continuous self-renewal.

What is fascinating in the intestine is that each cell inside the crypt, both precursor and differentiated, may acquire the ability to dedifferentiate, thus always guaranteeing the regeneration of the epithelium. This is the concept of intestinal plasticity that has emerged thanks to several genetic studies. Some authors have shown that the mobilization of the +4 cell pool is responsible for the repopulation of the stem cell niche inside the crypt [35,36,37], while others argue that short-lived secretory progenitors cells can dedifferentiate and acquire a bona-fide phenotype of LGR5+ cells to repopulate the crypt in case of injury [38,39]. Further studies have been focused on the ability of differentiated cells (secretory and absorptive) to return to stem cells conditions [40].

This matter is widely debated and seems that the niche environment can play an important role in the intestinal renewal providing the pivotal factors driving mature cells to reprogram their fate in case of necessity.

## 3. Disruptions of Intestinal Homeostasis: The Colorectal Cancer (CRC)

Despite the different architecture, the small and the large intestine share a high proliferating stem cell compartment at the crypt bottom. However, differently form the small intestine, cells of large intestine are more exposed to an inflammatory microenvironment due to different factors (stool compaction, waste substances, alteration in intestinal barrier permeability, and inflammatory diseases). This contributes to an increase tumor development in the large intestine, namely, CRC, with respect to the small one, for which the global incidence of tumor is low. Current knowledge defines CRC a step-wise tumor classified into four main subtypes based on gene expression profile, which differ from each other for genetic aberrations, signaling pathways, microenvironment features, and clinical outcomes [41,42].

Studies on the causes responsible for the CRC onset have established that it is linked to mutations in APC gene, a classical tumor suppressor gene. Sometimes, hereditary CRC are characterized by the loss of APC, a condition originally discovered in the familial adenomatous polyposis (FAP) subjects. Patients affected by FAP early develop a high number of adenomas and colon polyps, which subsequently progress into carcinoma. Genetically modified APC^Min/+^ mouse model resembling the FAP population has been generated to better understand the molecular mechanism leading to CRC development, which are based on mutations in APC gene [43,44,45]. Intestinal cells harboring DNA mutations, mainly regarding APC gene, are called cancer stem cells (CSC) and display an elevated proliferation rate, which finally results in the onset of CRC. A “top down” model of early stages of intestinal tumorigenesis have been so far described, in which the dysplastic cells at the top of the crypt are responsible for the initiation processes of the adenoma. These dysplastic cells also exhibit a mutation in APC gene [46]. This paradigm have been further challenged by a “bottom up” model, where APC mutations in CSC at the bottom of the crypt targeting WNT pathway lead to the expansion of intestinal stem cell compartment and to adenoma formation [47] (Figure 1B). Further studies have also shown that deletion of APC in LGR5+ stem cells remaining in the crypt bottom results in tumor growth and microadenoma development in 5 weeks. This may depend on the presence of the pool of Bmi-1 stem cells, which are a reservoir of stemness elements inside the crypt [48]. Additional studies proved that ISCs transformation can be due to the upregulated levels of the anti-apoptotic protein B-cell lymphoma 2 (BCL-2) targeted by the nuclear factor kappa-light-chain enhancer of activated B cells (NF-κB) pathway and highly express by LGR5+ CBC cells [49].

Behind the genetic alterations, inflammation represents a crucial factor for tumor development, as demonstrated by patients with inflammatory bowel disease (IBD): epidemiological studies indicate that a “high incidence” of CRC may arise from a pre-existing condition of IBD [50,51].

Importantly, hereditary cases of CRC, i.e., FAP patients, are infrequently preceded by inflammation events. In this case, the early events are characterized by mutations of the APC gene resulting in alteration of intestinal epithelial cell–cell junction and loss of enterocyte polarity favoring bacteria invasion and secretion of inflammatory cytokines, such as interleukins IL-23 and IL-17 [52]. As a result, other mutations can occur, including those of the oncogene KRAS and the gene encoding p53 (TP53). All these events result in the transformation of the adenoma into carcinoma.

In the development of colitis-associated CRC (CAC), mutations of p53 and other genes, as reported by exosome sequencing studies [53], occur and result in pro-inflammatory responses consisting in IL-6 cytokine production [54], the overexpression of tumor necrosis factor (TNF), and activation of reactive oxygen species (ROS) driving DNA damages. Therefore, inflammatory events and gene mutations together contributes to promote carcinoma onset [3].

Several evidence demonstrates an overall positive effect of anti-inflammatory therapy in reducing cancer rate and death in patients with IBD [3], thus suggesting an underlying interconnection between inflammation and CRC onset.

To understand the complex network behind the CRC development, several in vitro and in vivo studies have been conducted. However, it has been rapidly emerged how the in vitro analysis cannot recapitulate the great complexity at the basis of intestinal cancer. Indeed, it appears quite clear that not only enterocytes itself but a large set of cells, including the immune cells, are involved in the potential disruption of homeostasis that led to tumor onset.

## 4. LRH-1: An Ex-Orphan Nuclear Receptor

Liver Receptor Homolog 1 (LRH-1) is a nuclear receptor that belongs to NR5A or Ftz-F1 subfamily. Nuclear receptors are transcriptional factors involved in different physiological and pathological processes. They are structurally characterized by a variable N-terminal region, a conserved central DNA-binding domain (DBD), a variable hinge region, a conserved ligand-binding domain (LBD), and a variable C-terminal region. Thanks to these functional domains, nuclear receptors bind to specific region of the DNA and retain an extraordinary capability to be activated by a series of specific regulatory ligands, which finally promote the selective transcription of functional target genes [6].

Essentially expressed in endodermic tissues including intestine, liver, exocrine pancreas, and ovary, LRH-1 is involved in a series of physiological processes ranging from development to metabolism and steroidogenesis [7,55]. Differently from the most of nuclear receptors, which bind DNA as homodimers or heterodimers, LRH-1 can perform its transcriptional function binding to DNA as monomer through its unique and conserved Fitz-F1 DNA-binding domain [56]. Since its discovery in 1993 [57], several studies were conducted to elucidate LRH-1 structure and functions. Originally identified as an orphan nuclear receptor due to the unknown nature of its ligands, a few years ago, the crystal structure analysis revealed that phospholipids, including phosphatidyl inositol, can interact with LRH-1, functioning as its endogenous ligands [58,59,60]. Indeed, mutations to the LRH-1 ligand-binding pocket alter its phospholipid binding capacity, thus reducing its in vivo activity [61]. Besides endogenous ligands, LRH-1 can also be activated by several exogenous compounds. Lee et al. identified both dietary dilauroyl phosphatidylcholine (DLPC) and diundecanoyl phosphatidylcholine (DUPC) as powerful activators of LRH-1 in in vitro and in vivo models. By acting as regulator of lipid and glucose homeostasis, DLPC treatment decreases the amount of glucose, improves insulin sensitivity, and regulates triglycerides levels in two mouse model of insulin resistance [62]. Recently, Whitby et al. have developed a promising class of agonist of LRH-1 showing excellent regulation of target genes, which includes a bicyclic hexahydropentalene core scaffold, named “RJW100” [63,64]. In these years, thanks to the cutting-edge crystal structure studies, remarkable progresses have been made not only in the identification of specific exogenous agonists of LRH-1 but also in the comprehension of the exact interaction that takes place in the lipophilic ligand binding pocket, locking agonists in an aligned consistent orientation and potentiating their effects. In this regard, using differential scanning fluorimetry and cellular luciferase reporter assay, Mays et al. proposed a modification to the LRH-1 agonist scaffold RJW100, which significantly improves the binding mode, thus identifying the first low nanomolar LRH-1 agonist called 6N and providing an astonishing model of structure-guided medicinal chemistry approach [65].

LRH-1 plays a pivotal role in different biological processes, from proliferative, metabolic, and immunoregulatory activities to the early stage of development and cell commitment during differentiation [8]. As suggested from its name, the role of LRH-1 has been mainly elucidated in the liver, where it acts as the promoter-specific activator of cholesterol 7 alpha-hydroxylase (Cyp7a1), the first and rate-limiting enzyme of bile acid biosynthesis and cholesterol homeostasis [66,67,68,69]. Recently, close attention has been paid to the controversial role played by LRH-1 in intestinal homeostasis, regarding intestine cell proliferation and renewal as well as its involvement in intestinal inflammatory pathways.

### 4.1. LRH-1 in Stemness: A Boost for Cell Proliferation

LRH-1 performs a vital role in early development and differentiation, as clearly proved by embryonic lethality of LRH-1 null mice [70,71]. However, the LRH-1 activity is not only limited to the primordial phases of life. LRH-1 expression is preserved throughout the entire lifecycle and modulated during the various stages of development especially in tissue characterized by a high proliferative rate, such as those of the enterohepatic axis. Since the epiblast stage of embryonic growth, LRH-1 is essential to maintain Oct4 gene expression, the master regulator of pluripotency during the early stage of embryonic development, thus assuring the pluripotency of inner cell mass and embryonic stem cells [72,73,74]. Interestingly, LRH-1 can replace Oct4 in the derivation of induced pluripotent stem cells (iPSC) from murine somatic cells [75], underlying the pivotal role of LRH-1 in the control of development processes and cellular proliferation. In the primordial phase of organogenesis, LRH-1 is detectable in the yolk sac endoderm, branchial arch, and neural crest. Later, LRH-1 expression is preserved in the liver, in the pancreas, and in the intestinal crypts of Lieberkühn [76], contributing to define the enterohepatic phenotype.

Considering that homozygous LRH-1 knockout mice die before birth [70], the precise regulatory mechanism by which LRH-1 promotes cell proliferation and expansion growth of digestive organs has always been problematic to analyze. For this reason, Zhai et al. conducted a study on zebrafish, a useful model organism of developmental biology, demonstrating that the inactivation of LRH-1 results in hypoplastic endoderm organs. Indeed, the liver, the intestine, the endocrine, and the exocrine pancreas were significantly smaller in homozygous LRH-1 knockout (LRH-1^−/−^) larvae compared with control siblings. Notably, LRH-1^−/−^ fish showed that an increased number of cells stopped in G1 phase, while only a few cells were progressing in G2 phase, thus demonstrating that LRH-1 promotes G1 to S phase transition during cell proliferation. Specifically, LRH-1 induces the transcription of cyclin E1 (CcnE1) and acts as co-activator of WNT/β-catenin pathway, to promote the expression of cyclin D (CcnD1), thus regulating the critical G1-S phase transition. Furthermore, different genes that activate lipid metabolism were highly expressed in endoderm organs, suggesting that phosphatidylcholine signaling may activate LRH-1 to modulate the gene expression profile and the growth of digestive organs during zebrafish embryonic development [77].

**Figure 2:**
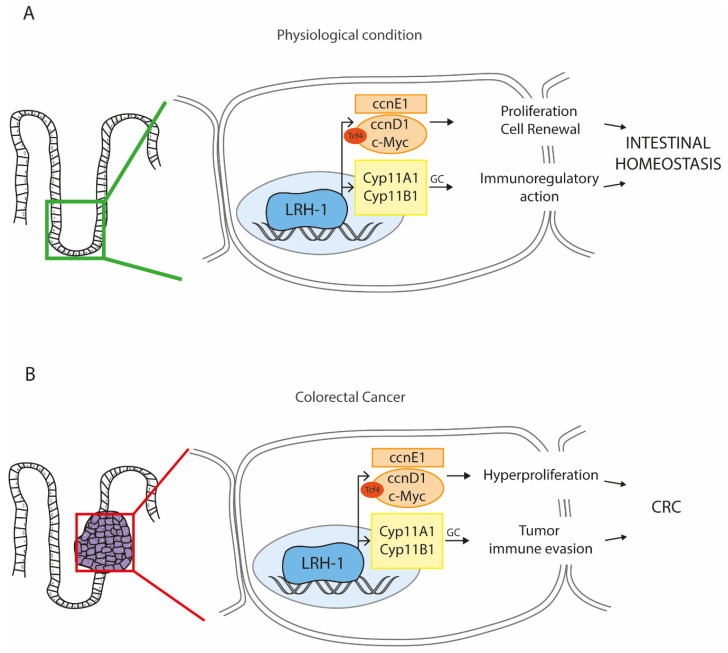
The multifaceted role of the Liver Receptor Homolog-1 (LRH-1) in the intestine. LRH-1 is nuclear receptor that can be endogenously activated by phospholipids present in the bile. (**A**) In physiological condition, LRH-1 is mainly expressed in the proliferative compartment of the intestine, where it drives the expression of genes involved in cell renewal and proliferation by regulating the WNT and NOTCH pathways, thus supporting the proper functions of stem cells. At the same time, LRH-1 can also regulate the intestinal glucocorticoids (GC) production, providing an overall immunoregulatory action. These two complementary functions of LRH-1 guarantee the intestinal homeostasis and counteract the onset of the inflammatory bowel disease (IBD). (**B**) The activation of LRH-1 in overt condition of colorectal cancer (CRC) may be deleterious. Indeed, the expression of the LRH-1 target genes results in hyperproliferation of cancer cells and tumor immune invasion, which collectively sustain the CRC progression (Abbreviations: ccnE1, cyclin E1; ccnD1, cyclin D1; Cyp11A1/B1, cytochrome P450 family 11 subfamily A/B member 1).

Besides embryogenesis, LRH-1 also plays a pivotal role in the perpetual renewal of the intestinal epithelium, consistently with its expression in the intestinal crypts [8]. Given the lethality of homozygous LRH-1 knockout mice [70], Botrugno et al. conducted a study in heterozygous LRH-1 knockout mice (LRH-1^+/−^) demonstrating that LRH-1 regulates intestinal cell renewal by controlling cell proliferation synergistically with β-catenin pathway [70]. Indeed, LRH-1 promotes cell cycle progression through two major mechanism: in a DNA binding-independent manner, by acting as a coactivator for β-catenin/Tcf4 to promote CcnD1 and c-Myc transcription, and in a DNA binding-dependent manner, in which LRH-1 itself directly binds the DNA and sustains CcnE1 transcription [70]. This dual transcription factor and co-activator role of LRH-1 convergently contributes to induce G1 cyclins synthesis, strengthening the LRH-1 involvement in cell cycle progression. More evidence highlighting the key importance of LRH-1 expression in the crypt compartment in order to maintain the homeostasis of the intestinal epithelium have been recently provided. Knocking out LRH-1 in the mouse intestine resulted in decreased Notch signaling, which, in turn, lowers the stem cell marker LGR5 and triggers apoptosis in the crypt [78]. This indicates that LRH-1 may play a role in epithelial cell differentiation in the intestine, behind ISC maintenance (Figure 2A). However, a detailed investigation of the exacted mechanism of action of LRH-1 in intestinal stemness is still far to be accomplished, but it would certainly shed a light into its contribution to physiological and pathological state.

### 4.2. LRH-1 in Inflammation: A Shield against Intestinal Bowel Diseases

Several lines of evidence support a critical role of LRH-1 in the control of intestinal immune homeostasis, mainly due to its contribution in the glucocorticoids’ synthesis. Anti-inflammatory glucocorticoids were originally thought to be produced only in the adrenal glands and secreted into the peripheral blood, where they affect the systemic immune responses. Nowadays, it is well known that endogenous glucocorticoids are produced also by extra-adrenal sources such as brain, thymus, skin, vasculature, and intestine [79]. The first evidence of intestinal production of glucocorticoids was reported in mice injected with anti-CD3 antibody, which lead to a strong activation of T cells. This robust immune alteration rapidly produced a counterregulatory protective response through intestinal strong upregulation of steroidogenic enzymes required for glucocorticoids synthesis. As demonstrated by in situ hybridization analysis, these enzymes are confined to the crypt region, suggesting that this induction may require crypt-enriched factors [80]. Notably, LRH-1 is highly expressed in the crypt where it finely exerts its intestinal immunoregulatory role triggering the expression of steroidogenic enzymes cholesterol side-chain cleavage enzyme P450scc (Cyp11A1), which converts cholesterol in pregnenolone, and 11β-hydroxylase (Cyp11B1), which finally converts deoxycorticosterone in corticosterone, the major glucocorticoid synthetized in mice [81]. Additionally, haploinsufficiency of LRH-1 renders mice more susceptible to experimentally induced colitis as demonstrated by increased necrotic regions and higher level of neutrophilic infiltration in the colon of LRH-1^+/−^ mice treated with 2,4,6-trinitrobenzene sulfonic acid (TNBS) or DSS, compared with control littermates. Notably, the colon of LRH1^+/−^ mice showed a lower local intestinal corticosterone production caused by an impaired intestinal expression of Cyp11A1 and Cyp11B1. According to this evidence, colon biopsies of patients with CD and UC reveal reduced expression of both LRH-1 and its steroidogenic target genes [82], therefore indicating that the activity of this NR is fundamental to keep gut homeostasis and counteract deleterious inflammation.

An interesting study conducted in human intestinal organoids, humanized murine intestinal organoids and a humanized murine IBD model demonstrated that LRH-1 promotes intestinal epithelial homeostasis and protects against intestinal inflammation. Considering that Notch expression in the intestinal crypt preserves LGR5+ stem cells [83], this study proved that acutely knocking out LRH-1 in mice decreases Notch signaling, thus increasing crypt cell death, which results in altered cellular configuration of the epithelium and modified epithelial barrier. On the other hand, the expression of human LRH-1 in the mouse intestinal epithelium relieved these alterations. Furthermore, overexpression of LRH-1 in both mouse and human intestinal organoids conferred epithelial resistance to both tumor necrosis factor-α (TNFα), a main pro-inflammatory cytokine in IBD, and 5-fluorouracil (5-FU), an intestinal toxic chemotherapeutic agent [78]. Intriguingly, the activation of LRH-1 by 6N agonist exhibits anti-inflammatory properties in humanized LRH-1 intestinal organoids exposed to TNF-α via upregulation of steroidogenesis [65]. However, whether the in vivo activation of LRH-1 by selective ligands would offer a potential therapeutic option for the IBD treatment remains to be addressed.

The heterologous intestinal environment combined with the different conditions that contribute to fine-tuning of the intestinal immune homeostasis should lead us to consider the intestinal immunological response not only as a matter of the single enterocyte but as a common action involving different cells type, including immune cells such as macrophages and T cells. For long time, the role of LRH-1 in immune cells has been widely ignored, partially due to its significantly lower expression levels in these cells compared with endodermal tissues. However, the first direct proof of LRH-1 expression in hematopoietic cells was presented by Lefèvre et al. that elucidate the role of LRH-1 in IL-13-induced macrophage differentiation and anti-infectious effector functions [84]. Further evidence demonstrate that LRH-1 regulates T cell expression of Fas (CD95) ligand (FASL), a critical T-cell effector molecule [85]. Recently, Seitz et al. demonstrated that LRH-1 deletion largely worsens T cell responses and antibody synthesis in vivo, resulting in the abrogation of CD4 T cell–mediated response in a mouse model of experimental colitis [86].

If on one hand, intestinal LRH-1 exerts a protective role against intestinal bowel disease due to its intestinal anti-inflammatory glucocorticoids’ synthesis promotion, then the other hand, LRH-1 in immune cells promotes T cell maturation, expansion, and effector functions, exacerbating T cell-mediated intestinal inflammatory diseases. The lower expression levels of LRH-1 in immune cells compared with intestinal tissue may shed a light in a possible therapeutic scenario in which low transient dose of LRH-1 inhibitors selectively delivered to immune cells could be helpful to contrast acute episodes of intestinal inflammation [87] without interfering with the highly expressed intestinal LRH-1, which in contrast confers an anti-inflammatory protection via its glucocorticoids synthesis function.

### 4.3. LRH-1 in Cancer: A Harmful Piece in the CRC Puzzle

In line with its crucial impact in inflammatory and proliferative pathways, increased evidence shed a light about LRH-1′s involvement in cancer. Until now, different tumors have been associated with an altered activity of this NR. LRH-1 is overexpressed in neoplastic pancreas compared to normal pancreatic tissues [88], as well as in non-small lung cancer cell carcinoma tissue compared with adjacent normal lung tissue [89]. LRH-1 is also abundantly expressed in breast carcinomas and positively correlated with in situ estrogen biosynthesis promotion [90,91]. Finally, LRH-1 is highly expressed in colon cancer tissues of CRC patients and its expression level is also significantly correlated with the overall survival (OS) rate of patients [92].

LRH-1 is considered a promotor of intestinal tumors’ formation by inducing G1 to S phase transition during cell proliferation due to a synergistic interaction with β-catenin. As previously described, LRH-1 enhances CcnE1 and CcnD1 expression by acting as a coactivator for β-catenin/Tcf4 to promote CcnD1 and c-Myc transcription. Moreover, LRH-1 can also directly bind to the cyclin E1 promoter, sustaining its transcription [70]. Considering the key determinant role played by WNT/β-catenin pathway in the self-renewal of intestinal cells, disruption of LRH-1 may drastically affect WNT/β-catenin signaling, leading to a selective proliferative advantage that promotes CRC onset and progression. Several studies were conducted to examine in depth the mechanism by which LRH-1 is involved in this scenario. Silencing of LRH-1 via shRNA in CRC cells line results in decreased cellular proliferation due to cell cycle arrest [93]. Additionally, overexpression of miR-374b alters WNT/β-catenin pathway and inhibits CRC cells proliferation and invasion through downregulation of LRH-1 expression [94]. In addition, miR-136, by targeting LRH-1, negatively modulates colon cancer proliferation and invasion in SW480 and HCT116 cell lines. On the other hand, miR-136 downregulation promotes overexpression of LRH-1 and aberrant activation of WNT/β-catenin signaling, thus sustaining intestinal tumorigenesis [95]. Furthermore, the siRNA-mediated LRH-1 knockdown in CRC cells inhibits cancer growth by transcriptionally repressing of p21 in a wild-type p53 background [96]. Inconsistently, LRH-1 silencing does not exert the same suppression in p53-mutated cell lines, therefore suggesting that the LRH-1 modulates different subsets of genes depending on the wider cellular context [96]. Recently, Lai et al. have demonstrated that by knocking down the expression of LRH-1 is possible to reduce the stemness-promoting effects of the transcriptional factor GATA-binding factor 6 (GATA6) in human CRC cell lines [97]. In a subsequent study, they also discovered that LRH-1 overexpression supports glycolysis in human CRC cells mostly by activating hypoxia-inducible factor 1α (HIF-1α). Interestingly, the lactate produced by glycolysis and up-taken via monocarboxylate transporter–1 (MCT-1) mediates the upregulation of peroxisome proliferator-activated receptor gamma coactivator-1 alpha (PGC-1α), a coactivator of LRH-1 [98], resulting in increased mitochondrial biogenesis and oxidative phosphorylation in LRH-1-overexpressing clones [99]. Although these results may appear conflicting, they underline a symbiotic mechanism existing between different subpopulation of CRC stem cells (CSC), where the lactate produced by the glycolytic/hypoxic population could be used by the oxidative population to sustain energy metabolism and intracellular redox homeostasis, thus sustaining the stemness proprieties of CSC [99]. Therefore, LRH-1 expression in CSC may sustain proliferation and cell renewal not only acting on molecular level by promoting cyclins expression but also on the metabolic processed. However, these data need to be confirmed also in vivo.

Sometime in vitro studies fail to replicate the complex, dynamic, and uncontrolled in vivo tumors’ micro-environment in which several intricates actors cooperate to cancer onset and progression. In this context, using both chemical and genetic mice model of CRC, Schoonjans et al. enlightened the tumor-supporting role of LRH-1. Specifically, in mice APC^Min/+^-based genetic colon cancer model, haploinsufficiency of LRH-1 blunts intestinal tumorigenesis. Similarly, in AOM chemical carcinogenesis model of mice colon cancer, haploinsufficiency of LRH-1 protects against aberrant colonic crypt foci formation [100]. These results suggest that LRH-1 promotes and exacerbates tumorigenesis in the colon. Considering that phospholipids act as LRH-1 endogenous ligands [59,60,61], Petruzzelli et al. focused on the role played by biliary phospholipids in intestinal regeneration and tumor progression [101]. In this regard, using Abcb4^−/−^ mouse model, which lacks biliary phospholipids secretion, they demonstrated a decreased colonic crypt length and cell proliferation in the absence of intraluminal biliary phospholipids, which was reverted by both treatment with LRH-1 agonist DLPC and diet enriched with phosphatidylcholine, therefore proposing a functional link between LRH-1 and phospholipids in the promotion of intestinal cell proliferation. Additionally, the absence of intraluminal biliary phospholipids protects Abcb4^−/−^ mice from intestinal tumorigenesis in both chemical and genetic AOM/DSS and APC^Min/+^ model of CRC. These findings highlight the crucial relevance of phospholipids-activated intestinal LRH-1 in promoting tumor progression, thus identifying this NR as a potential target for CRC [101].

The pro-tumoral and hyperproliferative role of LRH-1 in CRC deserves a substantial consideration, particularly regarding the controversial involvement of the LRH-1-mediated protective glucocorticoids synthesis in enterocytes. As we discussed earlier, the intestinal LRH-1 exerts an immunoregulatory role modulating immune cell-induced glucocorticoid synthesis via the induction of steroidogenic enzymes [81,82]. Tumor-infiltrating immune cells, such as T cells, macrophages and dendritic cells, release factors (i.e., TNFα, epidermal growth factor, etc.) are able to induce the activation of LRH-1 in CRC cells. Once activated, LRH-1 synthetizes glucocorticoids, which, in turn, negatively regulate the activation of tumor infiltrating immune cells, thus promoting the escape from cytotoxic mechanisms of destruction [102,103,104].

### 4.4. LRH-1 in the Gut: Connecting the Dots to Look Forward

In the previous paragraphs, we have remarked the crucial role enrolled by LRH-1 in both stemness and inflammation. These evidence has opened a window of fascinating possibility to study LRH-1 in both physiological and pathological intestinal scenario. Indeed, leading the expression of genes involved in proliferation and differentiation, LRH-1 sustains cell renewal of the proliferative compartment of the intestine, assuring a balanced homeostasis of the intestinal epithelium. Therefore, a proper activation of LRH-1 in different physiological conditions may sustain proliferation and direct a specific intestinal-lineage of intestinal stem cells, fixing possible intestinal dysfunctions and guaranteeing the proper operation of the gut. Remarkably, LRH-1 can also modulate the intestinal glucocorticoids production. Independently from their local immunoregulatory activities, glucocorticoids provide a contribution in induction of intestinal tight junction proteins [8,105]. For this reason, it could be interesting to investigate whether the stimulation or inhibition of intestinal LRH-1 activity may alter the epithelial barrier functions and intestinal permeability. Given the relevance of intestinal microbiota in affecting the development of either neuropsychiatric disorders (gut–brain axis) or liver disease (gut–liver axis), this capacity of LRH-1 would be of extreme importance for future research pointing in this direction. Lastly, LRH-1 is involved in intestinal inflammatory response by triggering the expression of steroidogenic enzymes, which finally promote the synthesis of corticosterone, thus counteracting IBD onset. This ability to control steroidogenesis may open novel perspective for the treatment of hormone-related disorders.

Further studies analyzing LRH-1 will be useful as a proof of concept to better understand the relevance of stemness and inflammation in the proliferation of intestinal cells and their contribution to intestinal cancer. It has already been examined the involvement of LRH-1 in different intestinal inflammatory condition as CD and UC. The extraordinary contribution of LRH-1 in intestinal proliferation and inflammation deserves a peculiar attention also in the pathological scenario of CAC, in which hyperproliferation and chronic inflammation represent the main cause of CRC onset. The intestinal role of LRH-1 itself is not to be considered singularly. If on one hand, LRH-1 stemness provides a physiological advantage in perpetual renewal and homeostasis, then on the other hand, it may represent one of the events that triggered the hyperproliferation of cancer cells. Indeed, the tumor immune evasion mediated by glucocorticoids synthetized by LRH-1 coupled together with the ability of this NR to maintain stemness proprieties of continue self-renewal and proliferation also in cancer cells may elect LRH-1 as a potential target in CRC therapy (Figure 2B). However, the patient’s history has to be taken in consideration in order to carefully establish most appropriate treatment and timing of administration. In the previous paragraphs, we reported that a pre-existent condition of IBD can be considered a powerful prerequisite to CRC onset. However, the intestinal LRH-1 activation in IBD subjects may ameliorate the disease state, by reducing inflammation and promoting the renewal of the damaged epithelia, thus limiting progression towards cancer. Nonetheless, given that LRH-1 in immune cells promotes T cell maturation, expansion, and effector functions, exacerbating T cell-mediated intestinal inflammatory diseases [87], it is important to selectively activate LRH-1 in the intestinal tissue to observe its beneficial effects. Indeed, an uncontrolled global activation may lead to a sustain intestinal inflammation, promoting the accumulation of mutagenic CAC-typical insults in cells residing in the crypt, conferring proliferative advantage, and favoring cancer onset. In this latter case, LRH-1 activation could be detrimental. As a matter of fact, LRH-1 agonists administration in cancer cell contribute to maintain a staminal phenotype, supporting the continuous proliferation and driving the spread of metastases [92,100]. Moreover, the stimulation of glucocorticoids production in this peculiar state can further worsen the tumor phenotype of overt form of CRC. Finally, since compelling evidence underline the role of LRH-1 in metabolism [99,101], also the metabolic state of the patients should be taken in consideration prior to proceed with an idealistic treatment. Noteworthy, a detailed knowledge of intestinal LRH-1 expression level and activity in IBD and CRC may contribute to define a clinical picture of the patients to elicit a specific targeted line treatment.

## 5. Conclusions

CRC is the main manifestation of a disrupted intestinal homeostasis, and colon CSC are the principal actors of this monstrous scenario. In normal condition, LRH-1 is involved in the maintenance of the intestinal epithelium, by stimulating the proliferation of the cells in the crypt compartment and by counteracting the harmful effects of inflammation via the production of glucocorticoids. However, the functions exerted by LRH-1 can be detrimental in an overt condition of CRC, where this NR sustains tumor growth and immune evasion. Indeed, LRH-1 expression has been negatively correlated with the overall survival in CRC. Although until now, only one clinical trial modulating LRH-1 activity has been carried out (ClinicalTrials.gov Identifier: NCT03481608), it would be intriguing to analyze the effect of LRH-1 agonists administration in IBD and CRC. Nevertheless, given the pro-metabolic role of LRH-1 in glucose homeostasis, this potential LRH-1 agonist treatment has to contemplate also the medical and metabolic patient history in order to pay the view of the personalized healthcare of CRC.

## Data Availability

Data sharing not applicable.
